# Resistance of a Rodent Malaria Parasite to a Thymidylate Synthase Inhibitor Induces an Apoptotic Parasite Death and Imposes a Huge Cost of Fitness

**DOI:** 10.1371/journal.pone.0021251

**Published:** 2011-06-16

**Authors:** Francis W. Muregi, Isao Ohta, Uchijima Masato, Hideto Kino, Akira Ishih

**Affiliations:** 1 Department of Infectious Diseases, Hamamatsu University School of Medicine, Hamamatsu, Japan; 2 Centre for Biotechnology Research and Development, Kenya Medical Research Institute (KEMRI), Nairobi, Kenya; 3 Laboratory for Ultrastructure Research, Research Equipment Centre, Hamamatsu University School of Medicine, Hamamatsu, Japan; 4 Institute of Experimental Animals, Hamamatsu University School of Medicine, Hamamatsu, Japan; Université Pierre et Marie Curie, France

## Abstract

**Background:**

The greatest impediment to effective malaria control is drug resistance in *Plasmodium falciparum*, and thus understanding how resistance impacts on the parasite's fitness and pathogenicity may aid in malaria control strategy.

**Methodology/Principal Findings:**

To generate resistance, *P. berghei* NK65 was subjected to 5-fluoroorotate (FOA, an inhibitor of thymidylate synthase, TS) pressure in mice. After 15 generations of drug pressure, the 2% DT (the delay time for proliferation of parasites to 2% parasitaemia, relative to untreated wild-type controls) reduced from 8 days to 4, equalling the controls. Drug sensitivity studies confirmed that FOA-resistance was stable. During serial passaging in the absence of drug, resistant parasite maintained low growth rates (parasitaemia, 15.5%±2.9, 7 dpi) relative to the wild-type (45.6%±8.4), translating into resistance cost of fitness of 66.0%. The resistant parasite showed an apoptosis-like death, as confirmed by light and transmission electron microscopy and corroborated by oligonucleosomal DNA fragmentation.

**Conclusions/Significance:**

The resistant parasite was less fit than the wild-type, which implies that in the absence of drug pressure in the field, the wild-type alleles may expand and allow drugs withdrawn due to resistance to be reintroduced. FOA resistance led to depleted dTTP pools, causing thymineless parasite death via apoptosis. This supports the tenet that unicellular eukaryotes, like metazoans, also undergo apoptosis. This is the first report where resistance to a chemical stimulus and not the stimulus itself is shown to induce apoptosis in a unicellular parasite. This finding is relevant in cancer therapy, since thymineless cell death induced by resistance to TS-inhibitors can further be optimized via inhibition of pyrimidine salvage enzymes, thus providing a synergistic impact. We conclude that since apoptosis is a process that can be pharmacologically modulated, the parasite's apoptotic machinery may be exploited as a novel drug target in malaria and other protozoan diseases of medical importance.

## Introduction

Malaria, caused by protozoan parasite of the genus *Plasmodium* is the most widespread parasitic disease, with malaria endemic regions encompassing approximately 40% of the global human population. Traditionally, four *Plasmodia* species cause human malaria, *P. falciparum*, *P. vivax*, *P. ovale* and *P. malariae*, but recently the primate parasite *P. knowlesi* was established as the fifth causative agent [1]. The global malaria situation is being exacerbated by the fact that *P. falciparum*, which causes about 90% of all global malaria cases, has rendered most of the classical antimalarials ineffective. More worrying are recent reports from Southeast Asia region, specifically near the Thai-Cambodia border indicating that resistance to the artemisinin-based combination therapies (ACTs), the only fully effective class of antimalarial drugs against falciparum malaria, is imminent [2]. Whereas the rising incidence in malaria morbidity and mortality is largely associated with drug failure following resistance, it is also possible that resistance induces an alteration of the intrinsic parasite traits that may influence parasite fitness (growth and multiplication) and virulence (harm to the host following infection), thus impacting on malaria mortality and morbidity [3], [4]. Drug pressure, the force that mainly drives antimalarial drug resistance through a population is a function of antimalarial drug use [5], [6]. While exposure of parasites to sub-curative drug doses facilitate the evolution of ‘classical drug resistance’ (point mutations, overexpression of target proteins), it can also engender genetically-encoded parasite traits that could influence parasite survival in a drug environment [7].

There are very few reports on how development of resistance in field populations of malaria parasites impacts on parasite fitness, and indeed studies on the fitness of drug-resistance genes of malaria parasites are in their rudimentary stage [8]. Therefore, parasite fitness and virulence owing to antimalarial drug resistance should be investigated since understanding how resistance in the field influences parasite fitness and pathogenicity can have a bearing on development of effective malaria control strategies. Unfortunately, this is extremely difficult to establish in the field especially in malaria endemic regions due to various confounding factors such as multiple clonal infections derived from separate infective bites [9]. We thus used a rodent model of malaria to generate resistance by exposing an isogenic strain of *Plasmodium berghei* to sub-therapeutic levels of the antifolates pyrimethamine (PYR) and 5-fluoroorotate (FOA) over several passages in mice. PYR inhibits dihydrofolate reductase (DHFR), thus depriving the parasite of essential folate cofactors. FOA is metabolized through the action of orotate phosphoribosyltransferase into 5-fluoro-orotidine 5′-monophosphate (5F-OMP), which in turn is converted into 5-fluoro-uridylate (5F-UMP) aided by orotidylate decarboxylase. 5F-UMP is further activated into 5-fluoro-2′-deoxyuridylate, known to be a potent inactivator of thymidylate synthase (TS), the obligate source of dTTP for DNA synthesis in malaria parasites since unlike the host cell, the parasite cell is unable to salvage preformed pyrimidines. Orotic acid is the only preformed pyrimidine that is utilized by malaria parasite, inspiring the belief that analogues of this substrate such as FOA can be toxic to the parasite [10], [11]. Whereas TS and DHFR in bacteria and metazoans occur as separate entities, that of malaria parasites and other protozoans occur as a TS-DHFR bipeptide coded off a single gene [12]. The bifunctional protein catalyzes formation of deoxythymidylate (dTMP) from deoxyuridylate (dUMP) in the thymidylate cycle using methelenetetrahydrofolate as a methylating agent. The dihydrofolate produced in this reaction is in turn reduced through DHFR catalysis to tetrahydrofolate [13]. Inactivation of TS is thus thought to be the primary mechanism through which FOA toxicity is mediated in malarial parasites. It has also been reported that an additional mechanism of FOA's toxicity may involve incorporation of fluorinated antimetabolites in form of 5-fluorouridine 5′-triphosphate (5F-UTP) into the RNA of malarial parasites [10].

In the present study, we observed that not only could a FOA-resistance line be readily generated, but also this resistance is stable and imposes a considerable loss of fitness to the resistant line. During growth in absence of drug, the FOA-resistant parasite line, but not the wild-type showed an apoptosis-like death. This observation is interesting since to the best of our knowledge, it is the first report where resistance to a chemical stimulus and not the stimulus itself is shown to induce apoptosis in a protozoan parasite. For a long time, apoptosis has been thought to be a preserve of metazoans, but mounting evidence within the last two decades demonstrating that programmed cell death (PCD) is also a feature of unicellular organisms is increasingly causing a paradigm shift on traditional tenets of PCD [14]. In metazoans, apoptosis represents a programmed form of cell death that plays a distinctive role in tissue development and homeostasis in response to an internal physiological disturbance [15]. However, apoptosis in unicellular organisms is thought to represent an altruistic behavior where a part of population which is less competent or non-viable is eliminated for the successful survival and onward transmission of the more competent organisms [16], [17]. So far, PCD has been described in diverse protozoan parasites including *Leishmania* [18], *Trypanasoma* [19], *Giardia* [20], in the ciliated protozoan *Tetrahymena thermophila* [21], *Entamoeba histolytica* [22], *Blastocystis hominis* [23] and *Plasmodia* [24]–[26]. PCD has also been reported in bacteria [27], in yeast [28], in the slime mould *Dictyostelium discoideum* [29], the dinoflagellate *Peridinium gatunense* [30], and in the euglenoid *Euglena gracilis* [31]. We report that irrespective of FOA-resistance mechanisms, the net biochemical effect in the aberrant parasite was depleted dTTP levels and its subsequent thymineless death through apoptosis.

## Materials and Methods

### Drugs, parasites and hosts, and ethics

Respective concentrations of 5-fluoroorotic acid hydrate (FOA) (Sigma®, USA) and pyrimethamine (PYR) (Sigma®, USA) were constituted by first solubilizing the compounds in dimethyl sulfoxide (final concentration <0.2%) and then dissolving in distilled water. The solutions were stored at 4°C until use. *Plasmodium berghei* (strain NK65), a rodent malaria parasite was used for all the studies. The parasite, maintained in a frozen state (−80°C) at the Parasite Bank of the Department of Infectious Diseases, Hamamatsu University School of Medicine was inoculated intraperitoneally (ip) into a male outbred ICR mouse, the donor mouse to the experimental mice. The day of infection was denoted as day 0 post-infection (pi), and all experiments were done using this revived parasite to ensure isogenicity of the parasite. Five days after parasite inoculation (at day 5 pi), its parasitaemia was assessed microscopically (Olympus BX50F4, Olympus Optical Co., LTD., Japan) at 1000× magnification by examining Giemsa-stained thin tail-vein blood smears, and its erythrocyte density was determined using a haemocytometer (F-520, Sysmex Corporation, Japan). The mouse was later sedated, bled via cardiac puncture and blood collected in heparinized tubes. The parasitaemia was adjusted downwards using physiological saline and each of the experimental male ICR mouse, 7-week old weighing about 30 g (Japan SLC Inc., Hamamatsu, Japan) was inoculated ip with approximately 1×10^5^ parasitisized erythrocytes in volumes of 0.2 ml [32]. The inoculated mice were randomized into appropriate groups, housed in cages and maintained in the animal facility on a commercial diet and water, *ad libitum*. This work was fully approved by the Institute of Experimental Animals, Hamamatsu University School of Medicine *(Approval Number: 2007087)*, in which facility the rodent models of this study were maintained and all animal experiments conducted, in accordance to the Institute's *Guide for the Care and Use of Laboratory Animals*. All mice that were either cured or were deemed to have completed their intended use were euthanased in the course of experiments.

### Exerting drug selection pressure to generate resistance

The ‘2% Relapse Technique’ (2% RT) of Peters and Robinson [33] was employed to exert drug selection pressure against *P. berghei* NK65 in mice, aiming to generate resistance. In this technique, a single dose of the test drug is administered to a group of mice immediately after infection, while a second group is infected but not treated to serve as a control. The drug dose is pre-determined such that it causes a delay time of between 5 and 7 days for the treated group to reach a 2% parasitaemia level, relative to the untreated controls. This delay time is termed as the ‘2% delay time’ (2% DT). The progress of infection is monitored daily and a new passage followed by immediate treatment is done on reaching the 2% DT. The acquisition of resistance is assessed by progressive reduction in 2% DT [33], [34]. In our study, 15 mice infected with *P. berghei* NK65 parasite (1×10^5^) were divided into three equal groups. The first two groups received oral doses (po) of 40 mg/kg bw. FOA and 30 mg/kg bw. PYR immediately after infection, while the third group served as the untreated control. We had earlier experimentally determined that these drug doses could cause a 2% DT of at least 5 days relative to the untreated controls. On attaining the 2% DT, the process of passaging and treatment was iterated over several generations while monitoring reduction in 2% DT.

### *In vivo* antimalarial assays to confirm resistance and its stability

Drug sensitivity studies can help determine the level of resistance of a generated ‘resistant line’ [33], [34]. To confirm resistance, the drug-exposed and the wild-type parasites were serial passaged in the absence of drug, and *in vivo* drug sensitivity studies undertaken at selected passages. For serial passaging, 6 mice per group were inoculated with respective parasites (1×10^5^) at day 0 pi and the parasitaemias of mice determined at day 7 pi, with one mouse per group serving as the parasite donor for subsequent passaging. Drug sensitivity studies were done at passages 2, 5, 10 and 12, where mice (in groups of 5) infected with respective parasites were treated po twice a day for three consecutive days starting from day 4 pi with either 6.67 mg/kg body weight (bw) FOA (40 mg/kg cumulative dose) or 5 mg/kg bw PYR (30 mg/kg cumulative dose). For each drug, at least two independent experiments were conducted. Drug efficacy was measured by percentage (%) parasitaemia suppression on day 7 pi after observation of Giemsa-stained thin blood smears under microscope, relative to the untreated controls; mice survival rate (%) and longevity relative to the untreated controls; and drug curative effect to mice (%). Mice that showed parasites on day 4 pi, but were aparasitaemic on subsequent days post-treatment up to day 60 pi were considered cured [35]. Percentage (%) parasitaemia suppression for the drugs was calculated as: 100−[(mean parasitaemia treated/mean parasitaemia control)×100] [36].

### Resistance cost of fitness

Growth and multiplication of a parasite can be used as a measure of its fitness, and since the parasites of this study are all isogenic (all were derived from the same wild-type) differing only on their drug responses, their growth rates was used as a measure of fitness [7], [37], [38]. Parasites growth rate between days 4 and 7 pi in the course of serial passaging in the absence of drug was used to assess the parasites proliferation rates for the FOA- and PYR-exposed parasites relative to their wild-type counterpart, and thus their fitness. The percentage loss of fitness of the mutant parasites relative to the wild-type was expressed as: 100−[(mean parasitaemia mutant/mean parasitaemia wild-type)×100]. For comparison of mean parasitaemias at different time points, parametric tests (two-tailed Student's t-test, one-way ANOVA) and non-parametric tests (Mann-Whitney, Kruskal-Wallis) were done using Microsoft Excel® 2004 and SPSS Statistics 17.0, respectively, with P<0.05 being considered significant.

### Morphological assessment of apoptosis through light and transmission electron microscopy

The morphologies of parasites previously subjected to selective drug pressure in mice were compared with that of the wild-type during their growth in the absence of drug through examination of Giemsa-stained thin tail-vein blood smears made at days 4 and 7 pi under light microscope. Giemsa-stained images were captured with a Live View Digital SLR Olympus E-620 camera (Olympus® Imaging Corp., Japan). The methods of Totino et al. [39] and Massimine et al. [40] were adapted for thin-section transmission electron microscopy (TEM), albeit with minor variations. The morphology of the FOA-resistant and the wild-type parasites were compared during passaging in the absence of drug. At day 7 pi, blood was collected in heparinized tubes as earlier described and used immediately for TEM studies. Parasitized erythrocytes were washed in phosphate buffered saline (PBS) and fixed with 2.0% glutaraldehyde in 0.1 M sodium cacodylate buffer (pH 7.2) for 1 h at 4°C, washed three times in the same buffer and fixed with 1% osmium tetroxide in 0.1 M cacodylate buffer for 1 h at room temperature. The cells were then washed in buffer, dehydrated in graded ethanol, and embedded in Epon. Ultrathin sections (50 to 60 nm thick) were cut using a Reichert ultramicrotome OmU3, collected on copper grids, double-stained with 2% aqueous uranyl acetate and lead citrate and observed in a JEM-1220 (JEOL Ltd., Japan) transmission electron microscope under 80 kV.

### Extraction of genomic DNA for oligonucleosomal DNA fragmentation analysis by polyacrylamide gel electrophoresis

Genomic DNA was extracted using the methods described previously [24], [41], albeit with some variations. In brief, blood was collected into heparinized tubes from mice infected with FOA-resistant or wild-type parasites at day 7 pi. Onto centrifuge tubes containing 4 ml Ficoll gradient solution (Ficoll-paqueTM PLUS), 2 ml of blood was gently layered at the top without mixing, and centrifuged at 1500 rpm for 30 min to separate erythrocytes from leukocytes, plasma and other constituents. The supernatant was aspirated and the pellets containing the red blood cells (RBCs) were washed with 1× PBS and centrifuged at 1800 rpm for 5 min, followed by aspiration of the supernatant. The pellets were aliquoted into eppendorf tubes, and RBC lysis buffer (0.15 M NH4Cl, 7 mM KHCO3, 1 mM EDTA) added in double volumes of the RBCs, thoroughly mixed and left to stand at room temperature for 10 min. The tubes were centrifuged at 8000 rpm for 5 min, and the supernatant discarded. The process of RBC lysis was repeated twice to ensure complete lysis. The pellets were suspended in 200 µl of cell lysis buffer (50 mM Tris pH 8.0, 20 mM EDTA, 1% SDS, and 0.1 M NaCl) to lyse their membranes, after which 20 µl of proteinase K (500 units/ml) and 10 µl RNase A (1 µg/µl) were added followed by incubation at 60°C in a thermomixer (at 300 rpm) for >1 h and cooled down at room temperature. An equal volume of phenol (in TE:10 mM Tris pH 8.0/1 mM EDTA, pH 8.0) was added to the tubes, gently mixed for 30 min and centrifuged at 15000 rpm for 15 min. The supernatant (nucleic acids) was transferred into clean eppendorf tubes and two new rounds of phenol protein extraction undertaken. The supernatant was again put into new eppendorf tubes and an equal volume of chloroform added, gently mixed for 5 min and centrifuged at 15000 rpm for 5 min. The supernatant was transferred into new tubes, 1/10 volumes of 3 M sodium acetate (pH 5.2) and 2.5 volumes of 99% ethanol were added to precipitate the DNA, and centrifuged at 15000 rpm for 5 min and ethanol aspirated. The DNA was washed with 70% ethanol, centrifuged again at 15000 rpm for 5 min and ethanol aspirated. The DNA was solubilized in 100 µl TE buffer and 2 µl RNase A added, followed by incubation at 37°C for 1 h in a thermomixer. The concentration of the DNA solution was determined by measurement of its absorbance at 260 nm by spectrophotometer. Total DNA (3 µ) was mixed with 10× BlueJuice gel loading buffer and tracking dye and loaded onto 8% polyacrylamide gel and electrophoresed in 1× TBE (Tris/Borate/EDTA) buffer for between 1 and 1.5 h at constant voltage of 120 V (maximum current 4 mA). The gel was then stained with ethidium bromide (1 µg/ml in water) for 30 min, rinsed with water and illuminated with ultraviolet light for examination and photography. Two independent experiments were conducted for nucleosomal DNA fragmentation analysis.

## Results

### FOA-resistance is readily generated in 100 asexual cycles

**Figure 1:**
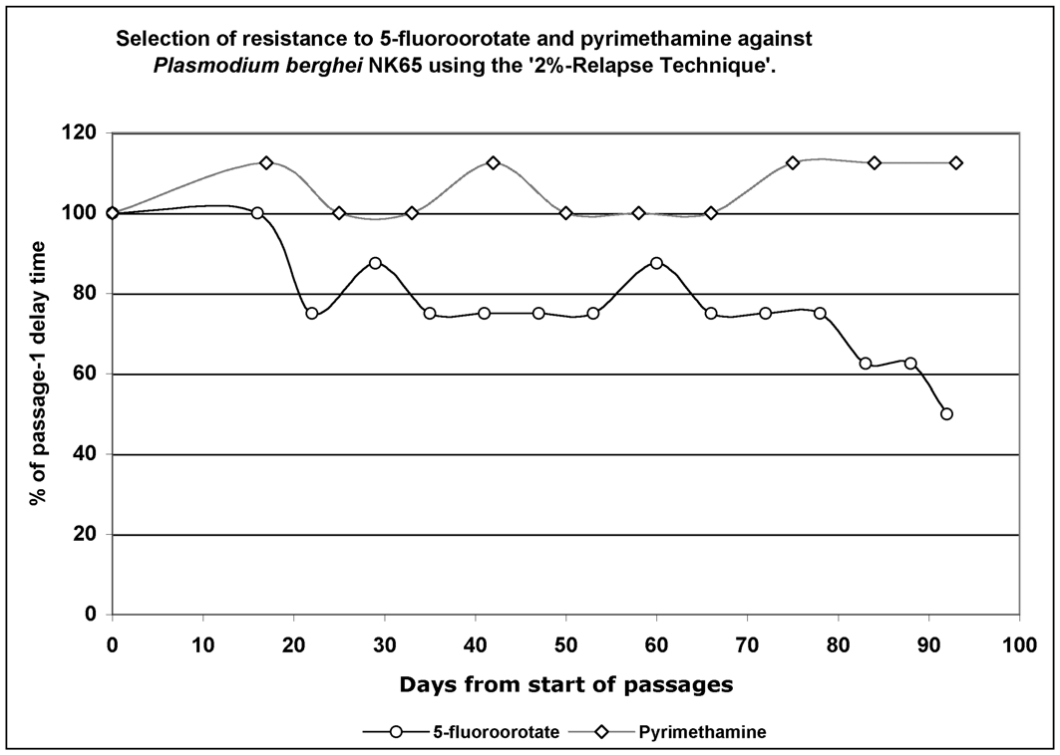
Generation of resistance using ‘2%-Relapse Technique’ (2% RT). In 12 serial passages lasting a total of 93 days, pyrimethamine-exposed parasite maintained a 2% DT of 8/9 days. In 15 serial passages, 5-Fluoroorotate parasite line (92 days) reduced its 2% DT from 8 days to 4, equalling the 2% DT for the wild-type parasite.

Figure 1 shows 2% DT of parasites subjected to FOA (40 mg/kg) and PYR (30 mg/kg) pressure in mice over successive serial passages, expressed as a percentage of 2% DT for passage-1. In 15 serial passages that lasted 92 days in presence of FOA, the 2% DT decreased by 50% from 8 days to 4, the time it took the wild-type parasite to achieve 2% parasitaemia in the absence of drug. The asexual blood stage cycle of *P. berghei* takes 22 to 24 h [42], and therefore FOA-resistance was generated in 92–100 parasite asexual cycles. However, parasites subjected to PYR over 12 serial passages (93 days, 93–101 asexual cycles) showed no change in 2% DT, which remained constant at 8 days.

### *In vivo* antimalarial assays confirmed that FOA-resistance was acquired, and that FOA-resistance is stable

**Figure 2:**
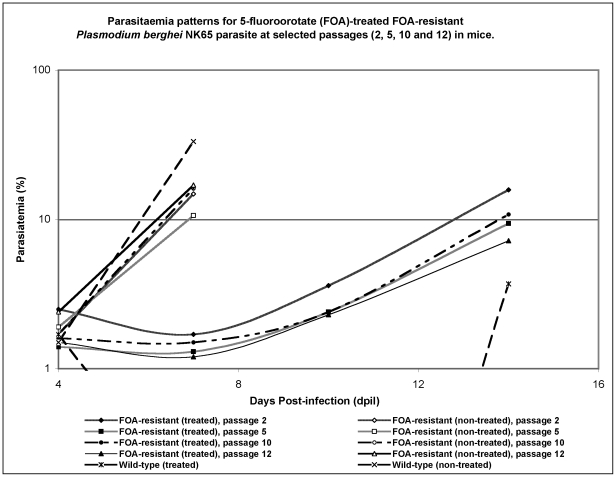
Parasitaemia patterns of mice infected with FOA-resistant parasite and orally treated with FOA. The mice were treated twice daily for 3 days with FOA (40 mg/kg cumulative dose) at passages 2, 5, 10 and 12. Note that the patterns mirror each other, confirming that the acquired FOA-resistance is stable. The discontinuous curve indicates that following FOA administration to mice infected with the wild-type parasite, no parasites could be observed under the microscope until day 14 p.i when recrudescent parasites were observed.

After subjecting parasites to selective drug pressure, another round of serial passaging in mice was initiated this time in the absence of the drug. FOA-exposed parasites were serially passaged for 12 generations that lasted over 72 days (72–79 cycles), while PYR-exposed and wild-type parasite lines were passaged for 10 generations. At passages 2, 5, 10 and 12, respective parasites lines were subjected to *in vivo* drug sensitivity studies with cumulative doses of 40 mg/kg FOA and 30 mg/kg PYR, administered po over 3 days from day 4 pi. Figure 2 shows the drug sensitivity patterns for FOA-exposed parasites at passages 2, 5, 10 and 12. Most of the untreated control mice infected with FOA-exposed, PYR-exposed and wild-type parasite lines died by day 10 pi with increasing parasitaemias. However, PYR-treated mice infected with either the PYR-exposed or wild-type parasites showed similar parasitaemia patterns at passages 2, 5 and 10, where no parasites could be observed under the microscope at days 7 and 10 pi, with recrudescent parasites appearing at day 14 pi (data not shown). The parasitaemia pattern for PYR-exposed parasite line mirrored that of the wild-type parasite (Figure 2). The observation that PYR-exposed line assumed the patterns of its wild-type counterpart is congruent with its unaltered 2% DT, confirming lack of resistance. On the other hand, mice infected with FOA-exposed parasite line and treated with FOA showed similar parasitaemia patterns (P>0.05) at passages 2, 5, 10 and 12, in which only a slight non-significant (P>0.05) decline in parasitaemia at day 7 pi was observed relative to day 4 pi, followed by gradual increase in parasitaemia. Comparison of parasitaemias at days 4 (before start of FOA treatment), 7, 10 and 14 pi across the four drug assays showed no statistical difference (P>0.05). This data is consistent with change in 2% DT for FOA-exposed parasite line and confirms that FOA-resistance was acquired, and that the resistance was stable. The stability of FOA resistance phenotype after a period of dormancy was also investigated. FOA-resistant parasites cryopreserved (−80°C) immediately after generation of resistance and stored for >1 year was revived in mice and assessed for resistance stability using *in vivo* antimalarial assays as described earlier. Parasitaemia patterns obtained after po treatment of mice infected with 10^5^ parasites with a cumulative dose of 40 mg/kg FOA over three days assumed the parasiatemia patterns of FOA-resistant line shown in Figure 2, indicating that resistance was not lost with dormancy (data not shown).

### FOA-resistance imposes a fitness cost to the parasite

**Figure 3:**
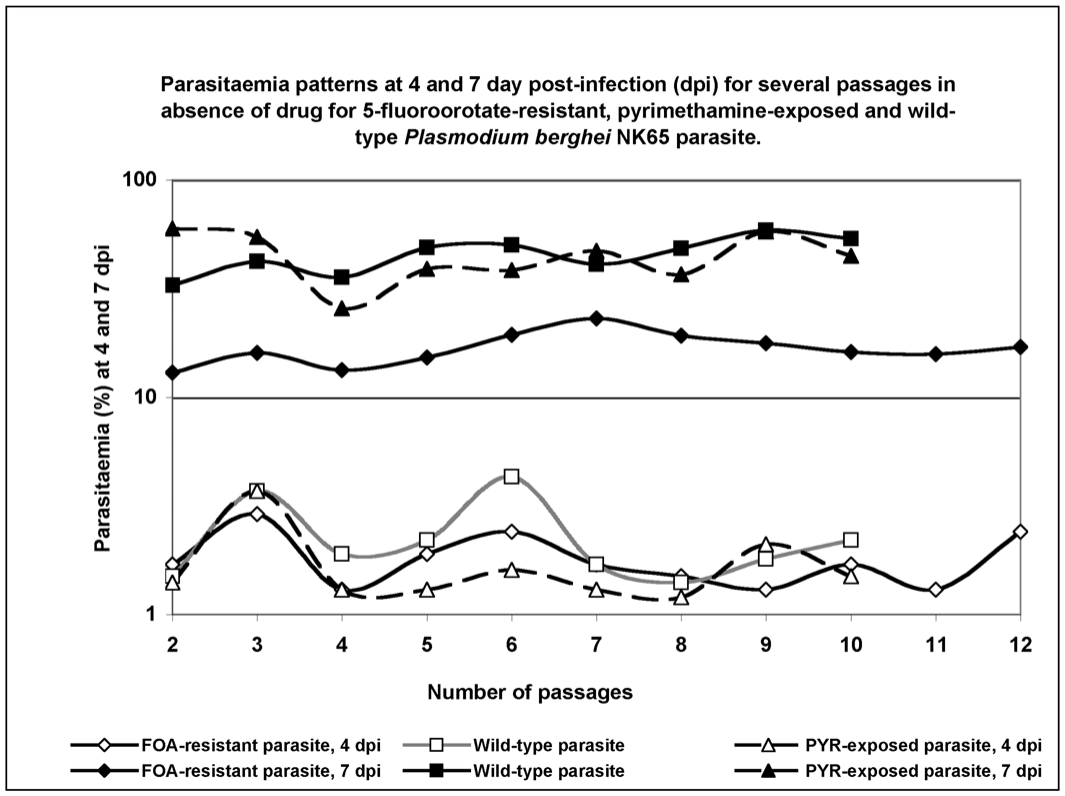
Comparison of growth patterns in the absence of drug for wild-type, FOA-resistant and PYR-exposed parasites. The parasiatemias were assessed at days 4 and 7 post infection (pi) in the course of serial passaging the respective parasite lines in mice for up to 12 serial passages in the absence of drug. The mean parasiatemias for the three parasite lines were not different (P>0.05) at day 4 p.i. At day 7 p.i, the wild-type parasite had 3 times faster growth rate than the FOA-resistant parasite. The PYR-exposed line showed >1.5-fold faster growth rates for the first three generations than the wild-type (P<0.05, day 7 p.i). However, at passage 4 (26 asexual cycles) the parasitaemia descended, and maintained a similar (P>0.05) growth pattern as that of the wild-type. Note that passage-2 is the first represented passage in the chart since the first passage (passage-1) is excluded since it served as a revival passage of the parasite in the donor mice.

Comparison of parasitaemias at days 4 and 7 pi among the three parasites lines (FOA-resistant, PYR-exposed and wild-type) grown in mice over 10–12 passages in the absence of drug (Figure 3) revealed that *altered intrinsic parasite properties influence its biological fitness*. Across the three parasites lines, there was no difference in parasitaemias at day 4 pi for each passage (P>0.05). However, at day 7 pi, there were clear and distinct patterns for each parasite line. The FOA-resistant line showed significantly low (P<0.05) growth rates over the 12 passages (mean parasitaemia 15.5%±2.9) relative to the wild-type parasite (45.6%±8.4). These differences in parasitaemias translate into a three-fold faster growth rate for the wild-type parasite, and an overall resistance cost of fitness of 66.0% for the FOA-resistant line. Interestingly, for the first three serial passages, the PYR-exposed parasite line had significantly higher parasitaemias than the wild-type parasite (P<0.05). Its mean parasitaemia of 59.7%±6.5 at passage-2 was approximately double that of the wild type (32.8±7.0%), indicating a two-fold faster growth rate than the latter. At passage 4 (after 26 days, 26 asexual cycles) the parasitaemia descended to the levels of the wild type, and maintained a similar (P>0.05) growth pattern for the subsequent passages. This implies that although a PYR-resistant phenotype could not be realized, the sustained PYR pressure had either altered the parasite genotypically or the parasite had acquired other intrinsic traits so as to cope with the new environment of drug pressure. Longevity of survival can also be used as an indicator of the harm a given parasite line imposes on the host. It was observed that mouse infected with the FOA-resistant parasite had slightly longer survival (12.0±3.5 days) which was statistically significant relative to the wild-type (P, 0.001) and PYR-exposed lines (P, 0.005) with survival of 8.9±0.7 and 9.3±0.8 days, respectively.

### Light and transmission electron microscopy reveals that resistance induces an apoptosis-like death in FOA-resistant parasite

**Figure 4:**
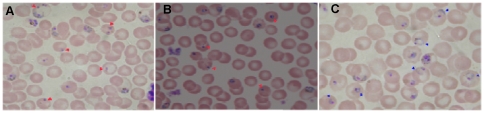
The photomicrographs of FOA-resistant and wild-type parasites during serial passaging in the absence of drug. A and B show FOA-resistant parasite at passages 2 and 12 respectively, with dark/condensed chromatin dot & cell shrinkage characterized by small and more dense cytosol (red arrowheads), confirming that the death phenomenon was a persistent feature of the FOA-resistant parasite line. The wild-type parasite (C) shows the typical ellipsoidal morphology and full cytoplasm.

**Figure 5:**
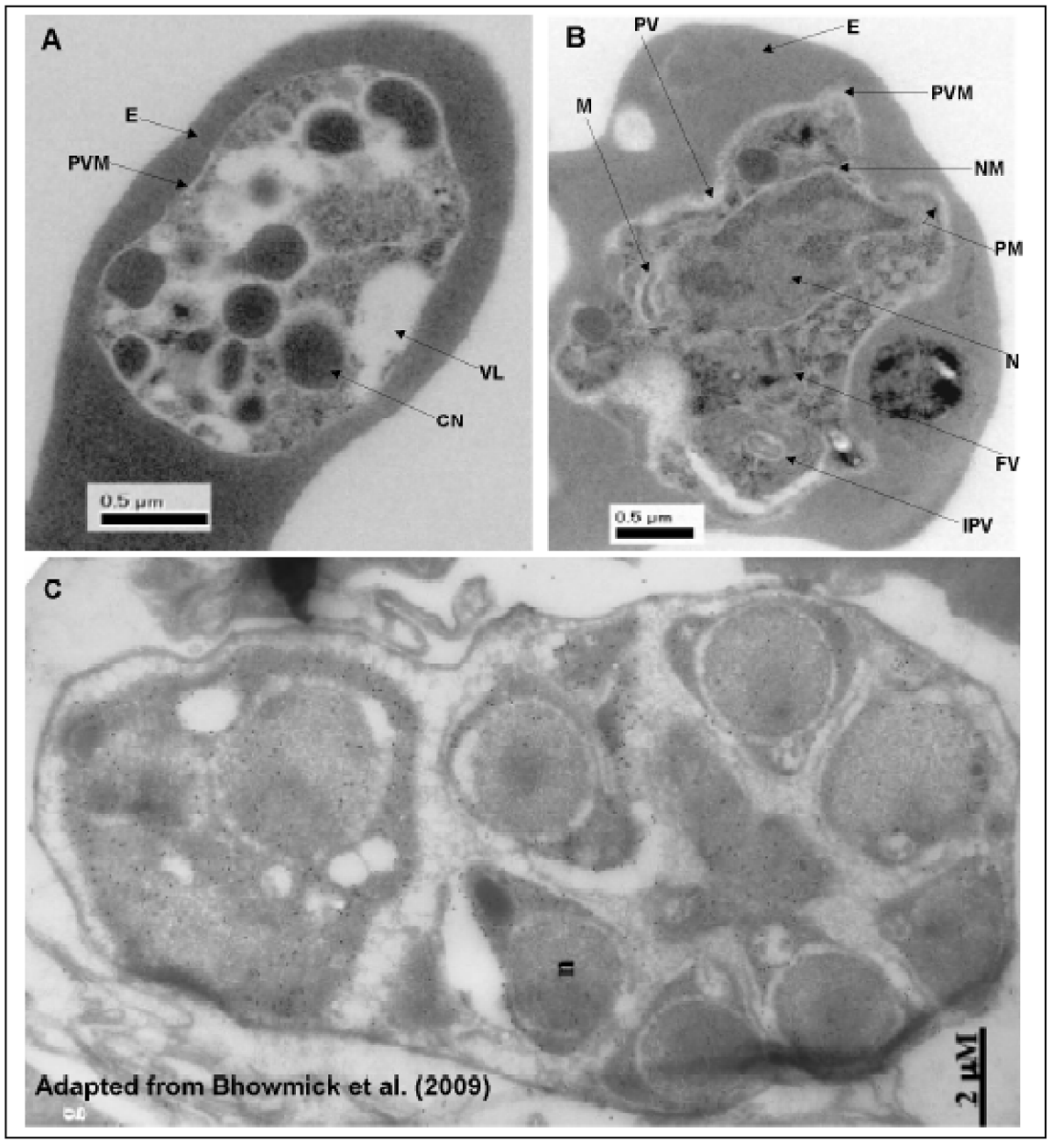
Electron micrographs of FOA-resistant and wild-type parasites grown in mice in the absence of drug. In (B), the trophozoite of the wild-type parasite shows normal ultrastructural morphology with various compartments and organelles well defined by membranes that have retained their integrity. In (A), late schizont of FOA-resistant parasite is seen with intact plasmalemma housing non-viable segmenters/merozoites that appear as electron dense bodies probably due to compaction of nuclear chromatin and condensation of cytoplasm. (C) is an immuno-gold electron micrograph adapted from Bhowmick et al. [114] showing a similar ‘syncytial’ cell from *P. falciparum* with viable merozoites. Note the distinct nucleus of segmenters. Abbreviations: CN, condensed segmenter; E, erythrocyte; FV, food vacuole; IPV, intraparasitic vacuole; M, mitochodria; N, nucleus; NM, nuclear membrane; PM, plasma membrane; PV, parasitophorous vacuole; PVM, parasitophorous vacuolar membrane; VL, vacuolization.

The morphologies of FOA-resistant and the wild-type parasites grown in the absence of drug in the course of serial passaging in mice were compared by light microscopic observation of thin blood smears. The FOA-resistant line showed abnormal parasites, the ‘crisis forms’ with decreased cytoplasmic volume and cell shrinkage, assumed to be parasites committed to death process. Other forms appeared as condensed bodies with darkly stained chromatin dots, assumed to be due to chromatin condensation, the classical morphological hallmark of apoptosis. As depicted in Figure 4 (A and B), the aberrant FOA-resistant parasite forms were observed in both the early and late phases of serial passaging. In contrast, the wild-type parasites showed normal ellipsoidal morphologies with normal chromatin dots and full cytoplasm (Figure 4C). To further confirm the apoptosis-like phenomenon observed in light microscopy, we conducted an ultrastructural examination of the wild-type and FOA-resistant parasite lines through TEM. During the early stage of parasite development (the ring forms and early trophozoites), there were no overt morphological differences between the wild-type and the FOA-resistant parasites that could set them apart. However, from mature to late trophozoites leading to schizonts, the FOA-resistant parasites showed pronounced structural differences from the wild-type line (Figure 5, A & B). The wild-type parasite retained its ultrastructural integrity with the nucleus, mitochondrion, digestive food vacuole and intraparasitic vacuoles showing well-defined morphologies. Furthermore, various membranes including the nuclear membrane and the parasitophorous vacuolar membrane fused with parasite plasma membrane appeared intact (Figure 5B). Figure 5A shows a markedly electron-lucent FOA-resistant parasite at late schizont stage, with electron-dense segmenters which appear to be non-viable. This TEM observation is consistent with the light microscopy (Figure 4A) where trophozoites and schizonts appear to be condensed.

### Resistance to FOA induces internucleosomal DNA fragmentation

**Figure 6:**
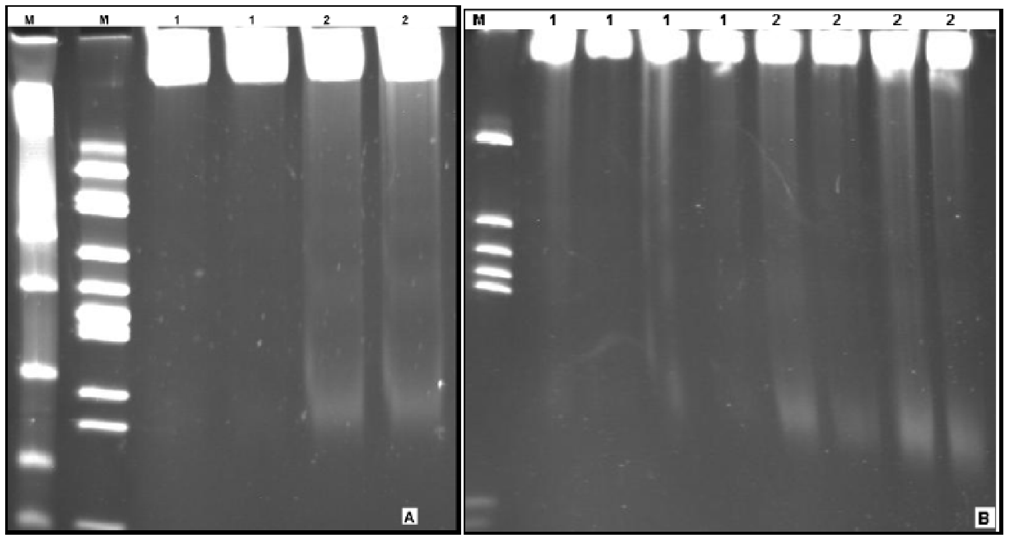
Resistance induces internucleosomal DNA fragmentation in FOA-resistant parasite grown in mice without drug. Polyacrylamide gel electrophoretic fraction of DNA was done in two independent experiments, A and B. In A, lanes 1 represent DNA extracted from the wild-type parasite that shows no cleavage, while lanes 2 represent cleaved DNA of FOA-resistant parasite, with the main band at ≈200 bp. Similar results are represented in B, where the wild-type shows no cleaved DNA (lanes 1), while FOA-resistant parasite shows cleaved DNA and a clear band at ≈200 bp (lanes 2). Lane M is the molecular size marker (pSG5/Hinf I and φX174/HincII for A, and pSG5/Hinf I for B).

DNA fragmentation was investigated using the genomic DNA extracted from FOA-resistant and wild-type parasites grown in the absence of drug. The DNA was subjected to polyacrylamide gel electrophoresis, which gives higher resolution for small molecules. In two independent experiments (Figure 6, A & B), we observed laddering patterns with the FOA-resistant but not the wild-type parasite, serving as clear evidence that apoptosis was a feature of FOA-resistant parasites since internucleosomal degradation of DNA is the biochemical hallmark of apoptosis. The sharpest DNA band was observed at ≈200 bp.

## Discussion

The impact of resistance on parasite fitness and disease severity (virulence) is a poorly understood phenomenon, much less the drug-specificity of these effects and thus warrants empirical investigations. To further knowledge on these aspects, we subjected a rodent malaria parasite to selective drug pressure using FOA and PYR, two antimetabolites that have specific targets and well-defined biochemical sequelae resulting from inhibition of their target enzymes. FOA-resistance was not only readily generated in ≈100 asexual cycles of exposure to FOA, but also it was stable (maintained over sequential blood-stage passages) as revealed by drug sensitivity studies (Figure 1 and 2). The stability was further confirmed by the observation that long periods of dormancy exceeding one year did not result in loss of resistance (data not shown). FOA is reported to be metabolized to toxic 5-fluoro-2′-deoxyuridylate which is a strong inhibitor of TS, an essential enzyme for the *de novo* synthesis of thymidylate and subsequently DNA synthesis. Since TS is the rate-limiting enzyme in the *de novo* pyrimidine biosynthesis, it serves as an excellent drug target [43]. FOA was previously reported to have potent *in vitro* antiplasmodial activity against *P. falciparum*, and to be curative in *in vivo* rodent models of malaria [44], [45].

Possible mechanisms via which FOA-resistance could be mediated include qualitative and/or quantitative aspects of the drug target through mutation, deletion or amplification. Other factors that limit the quantitative or temporal aspects of interaction between the drug and its target such as limitation of intracellular accumulation of the drug may also play a role in resistance [46]. In the absence of drugs or substrates, the TS of the mammalian cells binds to specific regions of its cognate mRNA prompting a direct feedback inhibition of TS mRNA translation [47]. It has been reported that similar translational control mechanism occurs in *Plasmodia* where TS-DHFR specifically blocks its own synthesis by binding to TS-DHFR mRNA [48]. It can therefore be expected that an aberrant TS is unable to bind to its cognate mRNA leading to overexpression of the TS enzyme, thus conferring resistance. If that were the case in FOA resistance, it would be expected that resistance due to overexpression of the TS domain of the TS-DHFR would also result in cross-resistance to antifolate drugs that target DHFR such as PYR, since DHFR domain would be overexpressed too. However in our study, the FOA-resistant parasite was equally susceptible to PYR (30 mg/kg) as the wild-type parasite (data not shown), and therefore resistance due to amplification of the *ts-dhfr* gene was ruled out. This is consistent with the fact that gene amplification of *ts-dhfr* gene has not been demonstrated in field isolates, and appears to play no role in clinical resistance [49]–[52]. In one-step *in vitro* selection protocol, Rathod et al. [53] were able to rapidly generate strong and genetically stable FOA-resistance (in 25 days) of up to 400-fold against *P. falciparum*. Also congruent with our study is the observation that FOA-resistance did not result in cross-resistance to the antifolate PYR. Events occurring upstream of TS like altered activation of FOA to 5-fluoro-orotidylate or altered means of transport of the drug may also contribute to FOA resistance. However, transport of FOA into the parasite cell is via tubovesicular membrane (TVM), which also transports a broad range of amino acid and nucleosides [54]–[56]. Thus, resistance through altered TVM would severely deprive the parasite of essential nutrients, thus hindering it growth and ability to complete normal cycle, a situation not observed in our study. In the light of the fact that FOA's primary target is the parasites TS [10], and that resistance to ‘single target’ antimalarial drugs such as PYR and atovaquone has consistently been shown to be mediated via point mutations on the genes of their target proteins [49], [50], [52], [57], [58], FOA resistance conferred by mutations is therefore plausible. Unlike FOA, PYR pressure over a similar period of time (≈100 asexual cycles) did not yield PYR resistance, as confirmed by drug sensitivity studies. This reinforces the already known fact that the rate of drug resistance, even for drugs with similar mode of action (both FOA and PYR are single-target drugs) is drug-specific.

When both FOA- and PYR-exposed parasite lines were subjected to several serial passages in the absence of the drug, it was observed that the effect of drug-exposure impacted their growth and multiplication capacities differently (Figure 3). The wild-type parasites showed a 3-fold faster growth rates than the FOA-resistant line over 12 serial passages, which translated to about 66% resistance cost of fitness. Interestingly, although the PYR-exposed line did not acquire the resistance phenotype, it showed a two-fold faster growth rate than the wild-type parasite during the first three serial passages, later assuming the latter's growth pattern which it retained for the subsequent serial passages. This implies that although the PYR-exposed line did not acquire the resistance phenotype and remained susceptible to PYR, either it was genotypically altered through mutations or had acquired other traits to enable it surmount the effects of drug pressure [7]. Resistance to antifolates is due to consecutive accumulation of point mutations on their target enzymes, mutations which act synergistically leading to drug failure [49]. In the case of PYR, the ser108asn mutation is established as the first step to PYR resistance, and initial mutations may not lead to quantifiable resistance. It is thus possible that due to PYR pressure, the PYR-exposed parasite acquired only the initial point mutations in its journey to become resistant.

Studies from a variety of pathogens including different species of bacteria, *Toxoplasma gondii*, and viruses indicate that mutations associated with drug resistance confer a fitness cost, and there seems to exist a general consensus that mutant forms of an organism are likely to be less fit than their wild-type strains in the absence of selection [59]–[68]. Whether or not drug-resistant mutants will survive in the absence of the drug is of clinical relevance because if the parasites harboring resistance genes are less fit than their wild-type counterparts, they would be expected to diminish in numbers relative to the sensitive forms in the absence of drug pressure. In such a scenario, the drug that generated the resistance could later be reintroduced for clinical use. Both field and laboratory reports regarding fitness costs of resistance in malaria parasites are controversial and ambivalent, although available data are largely skewed towards loss of fitness of the resistant mutants relative to the wild-type forms [69]–[84]. It has been reported that following CQ withdrawal in Malawi in 1993 after pervasive failure rate of up to 80%, prevalence of CQ-resistant *pfcrt* genotypes decreased from 85% (1992) to 13% (2000) [71]. These findings were corroborated by Mita et al. [72] who later established that this was due to expansion of the wild-type *pfcrt* allele in *P. falciparum* populations in absence of CQ pressure, rather than a back mutation of K76T to K76 [73]. Similar results have been reported from some areas of Southeast Asia including Hainan region of China, Thailand, and Vietnam following cessation of use CQ or SP [74]–[76], [79]–[81]. Hayward et al. [70] reported that in the absence of selective drug pressure, mutant *P. falciparum* strains harboring CQ-resistance *pfmdr1* alleles incurred a 25% fitness cost during the intraerythrocytic stage of growth.

Light microscopic examination of FOA-resistant and the wild-type parasites during serial passaging in the absence of drug revealed that the former had shrinking parasites with cytoplasm appearing condensed (more dense) (Figure 4, A and B) while the latter retained full ellipsoidal shape and normal cytoplasm (Figure 4C). It is noteworthy that FOA-resistant parasites consistently showed this morphology through the 12 serial passages undertaken. This observation was rendered further credence through TEM, where ultrastructures of both the wild-type and the FOA-resistant parasites were overtly different (Figure 5, A and B). The FOA-resistant parasite showed electron dense segmenters and merozoites possibly due to condensation of cytoplasm and compaction of nuclear chromatin, but within an intact plasmalemma (Figure 5A). In contrast, the wild-type parasite showed normal intracytoplasmic structures with well-defined membranes (Figure 5B). These observations are consistent with morphological characteristics of apoptosis. The hallmark biochemical feature of apoptosis is endonuclease-mediated DNA fragmentation, where DNA is initially cleaved into 300 kbp fragments and/or 50 kbp fragments [85], [86]. This is commonly (but not always) followed by further cleavage of DNA into oligonucleosomal-sized fragments due to double stranded cleavage of DNA at linker regions between nucleosomes, forming fragments of 180–200 bp and multimers thereof [15], [87]. In two different experiments, we observed cleaved DNA with a characteristic sharp band at ≈200 bp (Figure 6, A and B). Morphologic characteristics of cells undergoing apoptosis include cytoplasmic condensation, cell-shrinkage, nuclear condensation, DNA fragmentation and vacuolization, and preserved plasma membrane integrity with decreasing permeability [88], [14], [23]. In the late stages of apoptotic process, the dying cells fragment into smaller pieces known as apoptotic bodies, which are rapidly phagocytosed before membrane alterations can occur, and are subsequently degraded within lysosomes [15], [89].

How is apoptosis mediated in the FOA-resistant parasite cell? TS has been exploited in cancer chemotherapy, and is a target of fluoropyrimidines, as well as folate-based inhibitors including Tomudex®, Pemetrexed® and Nolatrexed® (Thymitaq) [44], [90]–[92]. The inhibition of TS results directly in depletion of dTMP and thus dTTP, an essential precursor for DNA leading to an accumulation of dUMP and thus dUTP. The dearth of dTTP in the cell leads to misincorporation of dUTP into DNA during DNA replication, prompting a DNA repair process involving dUTP excision by uracil-DNA glycosylase that leads to DNA damage and cell death, a phenomenon termed as thymineless cell death [44], [93], [94]. Thus, the imbalance in dTTP/dUTP and DNA damage can result in induction of downstream events leading to apoptosis [90], [95]. It is therefore plausible that a defective TS in FOA resistant mutants elicits similar sequelae to ones caused by chemical inhibition of the TS, leading to parasite death via apoptosis.

HL-60 cells represent an accepted classical model for apoptosis and thus using HL-60 as a standard [89], [96], it was estimated that the level of apoptosis observed in FOA-resistant parasites to be <10%, apparently a low level of parasite death to adequately account for observed huge fitness cost of 66%. However, the early cellular events in apoptosis are accomplished quickly, with only a few minutes elapsing between onset of the process and the formation of a cluster of apoptotic bodies. The subsequent digestion of the phagocytosed apoptotic bodies is also rapid and completed in less than an hour that even when there is a large-scale PCD, only few dead cells can be observed [85], [97]–[99]. Thus, it has been argued that due to rapidity of apoptotic events *in vivo*, relatively small fraction of apoptotic cells observed represents a considerable cumulative cell death [15], [100], [101]. Moreover, the development of blood stages of *P. berghei* in rodent models is asynchronous, and since nucleic acids syntheses in malaria parasite largely occurs at the trophozoite stage, it can only be expected that parasite cells will show varying degree of apoptosis depending on their phase of growth [11], [102]–[106]. Finally, it is possible that since the asexual stage parasite largely resides in the erythrocyte with which it has close interactions (eg. through TVM networks), the death of a ‘housed’ parasite could spawn rapid eryptosis [107]–[109] thus making it difficult to quantify apoptosis in blood stage parasites.

To date, not more than three studies have demonstrated that asexual stages of *P. falciparum cultures* undergo chemically induced apoptosis. Picot et al. [24] were the first to demonstrate plasmodial apoptosis after observing oligonucleosomal DNA fragmentation in CQ-sensitive (3D7) *P. falciparum* cultures exposed to CQ. This was corroborated by Deponte and Becker [110], and later by Meslin and co-workers [25] who reported that *P. falciparum* cultures exposed to various chemicals had typical features of apoptosis including internucleosomal fragmentation and disruption of mitochondrial membrane potential. It has also been reported that sexual stages of malaria parasite also undergo apoptosis during sporogonic development in the mosquito vector as a means of regulating parasite density. Al-Olayan et al. [26] observed that during development in the *Anopheles stephansi* midgut, *P. berghei* exhibited features of typical apoptosis found in metazoans including chromatin condensation, DNA fragmentation and externalization of phosphotidylserine to the outer lamellae of the cell membrane. Interestingly, all the apoptotic features were replicated *in vitro*, an indication that apoptosis plays a pivotal role in controlling sporogony [26]. In the present study, we demonstrate for the first time that *in vivo*, blood stage asexual forms of *malaria* parasites can undergo drug resistance-induced apoptosis. Although unicellular eukaryotes lack caspases—the executioners of apoptosis in metazoans—there is now mounting evidence that members of cysteine peptidases clan CD may be functionally homologous to metazoan caspases and could be involved in a less regulated form of apoptosis in single-celled eukaryotes [111]. Genes encoding two cysteine proteases, the metacaspase-like proteins, PF14_0363 and PF13_0289 were identified from the *Plasmodium* genome database although their function in mediating apoptosis remains to be characterized [25], [112].

It is apparent that protozoan parasites have evolved effectors and regulators of PCD different from that of multicellular organisms, suggesting that the process of cell suicide in unicellular organisms may be harnessed as a therapeutic strategy to selectively activate PCD in the parasites [16]. Apoptotic machinery can be stimulated or inhibited using either internal or external stimuli including drugs. Therefore, a greater understanding of the effector and regulatory mechanisms of PCD in malaria and other protozoan parasites should be pursued to aid in rational design and development of novel selective therapeutics targeted to the specific biomolecules of the parasite. Encouragingly, this has a precedent in cancer therapy, since anticancer agents are known to mediate their activity through apoptosis [113]. To the best of our knowledge, this is the first *in vivo* report to show that resistance to a chemical (drug) other than the drug itself can induce apoptosis in malaria parasite. Today, there is no reliable system to reproducibly study PCD in malaria. We thus offer FOA-resistant parasite as a biological tool to help validate and understand the molecular basis underlying apoptosis not only in malaria parasites, but also in other protozoan parasites of medical importance. Several anticancer agents target TS and mechanism of resistance to TS antimetabolites as well as the subsequent biochemical sequelae may be similar to that of FOA-resistant malaria parasites. If this can be demonstrated to be true, then our findings have potential to be exploited in cancer therapy where apoptosis of TS inhibitor-resistant tumour cells can be optimized by chemotherapeutically targeting pyrimidine salvage enzymes such as thymidine kinase, thus providing a synergistic impact. We conclude that since apoptosis is a process that can be pharmacologically regulated, the malaria parasite's apoptotic machinery may itself be exploited as a novel drug target and/or could aid in identifying new targets for therapeutic intervention.

## References

[1] Schlitzer M (2007). Malaria chemotherapeutics part I: History of antimalarial drug development, currently used therapeutics, and drugs in clinical development.. Chem Med Chem.

[2] Noedl H, Se Y, Schaecher K, Smith BL, Socheat D (2008). Evidence of artemisinin-resistant malaria in western Cambodia.. N Engl J Med.

[3] Grech K, Watt K, Read AF (2006). Host-parasite interactions for virulence and resistance in a malaria model system.. J Evol Biol.

[4] Mackinnon MJ, Read AF (2004). Immunity promotes virulence evolution in a malaria model.. PLoS Biol.

[5] Hastings IM, Watkins WM (2006). Tolerance is the key to understanding antimalarial drug resistance.. Trends Parasitol.

[6] Gatton ML, Hogarth W, Saul A (2001). Time of treatment influences the appearance of drug-resistant parasites in *Plasmodium falciparum* infections.. Parasitology.

[7] Schneider P, Chan BH, Reece SE, Read AF (2008). Does the drug sensitivity of malaria parasites depend on their virulence?. Malar J.

[8] Walliker D (2005). The hitchhiker's guide to malaria parasite genes.. Trends Parasitol.

[9] Hastings IM (2003). Malaria control and the evolution of drug resistance: an intriguing link.. Trends Parasitol.

[10] Rathod PK, Leffers NP, Young RD (1992). Molecular targets of 5-fluoroorotate in the human malaria parasite, *Plasmodium falciparum*.. Antimicrob Agents Chemother.

[11] Gutteridge WE, Trigg PI (1970). Incorporation of radioactive precursors into DNA and RNA of *Plasmodium knowlesi in vitro*.. J Protozool.

[12] Yuvaniyama J, Chitnumsub P, Kamchonwongpaisan S, Vanichtanankul J, Sirawaraporn W (2003). Insights into antifolate resistance from malarial DHFR-TS structures.. Nat Struct Biol.

[13] Ivanetich KM, Santi DV (1990). Bifunctional thymidylate synthase-dihydrofolate reductase in protozoa.. Faseb J.

[14] Villalba JD, Gomez C, Medel O, Sanchez V, Carrero JC (2007). Programmed cell death in *Entamoeba histolytica* induced by the aminoglycoside G418.. Microbiology.

[15] Allen RT, Hunter WJ, Agrawal DK (1997). Morphological and biochemical characterization and analysis of apoptosis.. J Pharmacol Toxicol Methods.

[16] Welburn SC, Barcinski MA, Williams GT (1997). Programmed cell death in trypanosomatids.. Parasitol Today.

[17] Baton LA, Warr E, Hoffman SA, Dimopoulos G, Manuel J, Martin P (2008). Programmed cell death during malaria parasite infection of the vertebrate host and mosquito vector. Programmed Cell Death in Protozoa.

[18] Holzmuller P, Sereno D, Cavaleyra M, Mangot I, Daulouede S (2002). Nitric oxide-mediated proteasome-dependent oligonucleosomal DNA fragmentation in *Leishmania* amazonensis amastigotes.. Infect Immun.

[19] De Souza EM, Menna-Barreto R, Araujo-Jorge TC, Kumar A, Hu Q (2006). Antiparasitic activity of aromatic diamidines is related to apoptosis-like death in *Trypanosoma cruzi*.. Parasitology.

[20] Chose O, Sarde CO, Noel C, Gerbod D, Jimenez JC (2003). Cell death in protists without mitochondria.. Ann N Y Acad Sci.

[21] Kobayashi T, Endoh H (2003). Caspase-like activity in programmed nuclear death during conjugation of *Tetrahymena thermophila*.. Cell Death Differ.

[22] Ramos E, Olivos-Garcia A, Nequiz M, Saavedra E, Tello E (2007). *Entamoeba histolytica*: apoptosis induced *in vitro* by nitric oxide species.. Exp Parasitol.

[23] Nasirudeen AM, Hian YE, Singh M, Tan KS (2004). Metronidazole induces programmed cell death in the protozoan parasite *Blastocystis hominis*.. Microbiology.

[24] Picot S, Burnod J, Bracchi V, Chumpitazi BF, Ambroise-Thomas P (1997). Apoptosis related to chloroquine sensitivity of the human malaria parasite *Plasmodium falciparum*.. Trans R Soc Trop Med Hyg.

[25] Meslin B, Barnadas C, Boni V, Latour C, De Monbrison F (2007). Features of apoptosis in *Plasmodium falciparum* erythrocytic stage through a putative role of PfMCA1 metacaspase-like protein.. J Infect Dis.

[26] Al-Olayan EM, Williams GT, Hurd H (2002). Apoptosis in the malaria protozoan, *Plasmodium berghei*: a possible mechanism for limiting intensity of infection in the mosquito.. Int J Parasitol.

[27] Sat B, Hazan R, Fisher T, Khaner H, Glaser G (2001). Programmed cell death in *Escherichia coli*: some antibiotics can trigger mazEF lethality.. J Bacteriol.

[28] Madeo F, Frohlich E, Ligr M, Grey M, Sigrist SJ (1999). Oxygen stress: a regulator of apoptosis in yeast.. J Cell Biol.

[29] Cornillon S, Foa C, Davoust J, Buonavista N, Gross JD (1994). Programmed cell death in *Dictyostelium*.. J Cell Sci.

[30] Vardi A, Berman-Frank I, Rozenberg T, Hadas O, Kaplan A (1999). Programmed cell death of the dinoflagellate *Peridinium gatunense* is mediated by CO(2) limitation and oxidative stress.. Curr Biol.

[31] Scheuerlein R, Treml S, Thar B, Tirlapur UK, Hader DP (1995). Evidence for UV-B-induced DNA degradation in *Euglena gracilis* mediated by activation of metal-dependent nucleases.. J Photochem Photobiol B.

[32] Muregi FW, Kino H, Ishih A (2008). *Plasmodium berghei*: lack of antimalarial activity of an analogue of folate precursor, 2,4-diamino-6-hydroxymethylpteridine in a mouse model.. Exp Parasitol.

[33] Peters W, Robinson BL (1999). The chemotherapy of rodent malaria. LVI. Studies on the development of resistance to natural and synthetic endoperoxides.. Ann Trop Med Parasitol.

[34] Peters W, Robinson BL (2000). The chemotherapy of rodent malaria. LVIII. Drug combinations to impede the selection of drug resistance, Part. 2: The new generation–artemisinin or artesunate with long-acting blood schizontocides.. Ann Trop Med Parasitol.

[35] Benoit-Vical F, Lelievre J, Berry A, Deymier C, Dechy-Cabaret O (2007). Trioxaquines are new antimalarial agents active on all erythrocytic forms, including gametocytes.. Antimicrob Agents Chemother.

[36] Muregi FW, Ishih A, Suzuki T, Kino H, Amano T (2007). *In vivo* antimalarial activity of aqueous extracts from Kenyan medicinal plants and their chloroquine (CQ) potentiation effects against a blood-induced CQ-resistant rodent parasite in mice.. Phytother Res.

[37] Giha HA, Elbashir MI, A-Elbasit IE, A-Elgadir TME, ElGhazali GE (2006). Drug resistance-virulence relationship in *Plasmodium falciparum* causing severe malaria in an area of seasonal and unstable transmission.. Acta Trop.

[38] Stein WD, Sanchez CP, Lanzer M (2009). Virulence and drug resistance in malaria parasites.. Trends Parasitol.

[39] Totino PR, Daniel-Ribeiro CT, Corte-Real S, de Fatima Ferreira-da-Cruz M (2008). *Plasmodium falciparum*: erythrocytic stages die by autophagic-like cell death under drug pressure.. Exp Parasitol.

[40] Massimine KM, McIntosh MT, Doan LT, Atreya CE, Gromer S (2006). Eosin B as a novel antimalarial agent for drug-resistant *Plasmodium falciparum*.. Antimicrob Agents Chemother.

[41] Cheng Q, Lawrence G, Reed C, Stowers A, Ranford-Cartwright L (1997). Measurement of *Plasmodium falciparum* growth rates *in vivo*: a test of malaria vaccines.. Am J Trop Med Hyg.

[42] Hall N, Karras M, Raine JD, Carlton JM, Kooij TW (2005). A comprehensive survey of the *Plasmodium* life cycle by genomic, transcriptomic, and proteomic analyses.. Science.

[43] Van Triest B, Pinedo HM, Giaccone G, Peters GJ (2000). Downstream molecular determinants of response to 5-fluorouracil and antifolate thymidylate synthase inhibitors.. Ann Oncol.

[44] Rathod PK, Khatri A, Hubbert T, Milhous WK (1989). Selective activity of 5-fluoroorotic acid against *Plasmodium falciparum in vitro*.. Antimicrob Agents Chemother.

[45] Rathod PK, Gomez ZM (1991). *Plasmodium yoelii*: oral delivery of 5-fluoroorotate to treat malaria in mice.. Exp Parasitol.

[46] Fisher TC, Milner AE, Gregory CD, Jackman AL, Aherne GW (1993). bcl-2 modulation of apoptosis induced by anticancer drugs: resistance to thymidylate stress is independent of classical resistance pathways.. Cancer Res.

[47] Chu E, Voeller DM, Morrison PF, Jones KL, Takechi T (1994). The effect of reducing reagents on binding of thymidylate synthase protein to thymidylate synthase messenger RNA.. J Biol Chem.

[48] Zhang K, Rathod PK (2002). Divergent regulation of dihydrofolate reductase between malaria parasite and human host.. Science.

[49] Cowman AF, Morry MJ, Biggs BA, Cross GA, Foote SJ (1988). Amino acid changes linked to pyrimethamine resistance in the dihydrofolate reductase-thymidylate synthase gene of *Plasmodium falciparum*.. Proc Natl Acad Sci U S A.

[50] Peterson DS, Walliker D, Wellems TE (1988). Evidence that a point mutation in dihydrofolate reductase-thymidylate synthase confers resistance to pyrimethamine in falciparum malaria.. Proc Natl Acad Sci U S A.

[51] Gregson A, Plowe CV (2005). Mechanisms of resistance of malaria parasites to antifolates.. Pharmacol Rev.

[52] Foote SJ, Galatis D, Cowman AF (1990). Amino acids in the dihydrofolate reductase-thymidylate synthase gene of *Plasmodium falciparum* involved in cycloguanil resistance differ from those involved in pyrimethamine resistance.. Proc Natl Acad Sci U S A.

[53] Rathod PK, Khosla M, Gassis S, Young RD, Lutz C (1994). Selection and characterization of 5-fluoroorotate-resistant *Plasmodium falciparum*.. Antimicrob Agents Chemother.

[54] Lauer SA, Rathod PK, Ghori N, Haldar K (1997). A membrane network for nutrient import in red cells infected with the malaria parasite.. Science.

[55] Akompong T, VanWye J, Ghori N, Haldar K (1999). Artemisinin and its derivatives are transported by a vacuolar-network of *Plasmodium falciparum* and their anti-malarial activities are additive with toxic sphingolipid analogues that block the network.. Mol Biochem Parasitol.

[56] Haldar K, Akompong T, Rosenthal PJ (2001). Transport and Trafficking in *Plasmodium*-Infected Red cells.. Antimalarial Chemotherapy: Mechanisms of Action, Resistance, and New Directions in Drug Discovery.

[57] Srivastava IK, Morrisey JM, Darrouzet E, Daldal F, Vaidya AB (1999). Resistance mutations reveal the atovaquone-binding domain of cytochrome b in malaria parasites.. Mol Microbiol.

[58] Korsinczky M, Chen N, Kotecka B, Saul A, Rieckmann K (2000). Mutations in *Plasmodium falciparum* cytochrome b that are associated with atovaquone resistance are located at a putative drug-binding site.. Antimicrob Agents Chemother.

[59] Schrag SJ, Perrot V (1996). Reducing antibiotic resistance.. Nature.

[60] Levin BR, Perrot V, Walker N (2000). Compensatory mutations, antibiotic resistance and the population genetics of adaptive evolution in bacteria.. Genetics.

[61] Bjorkman J, Hughes D, Andersson DI (1998). Virulence of antibiotic-resistant *Salmonella typhimurium*.. Proc Natl Acad Sci U S A.

[62] Bjorkman J, Andersson DI (2000). The cost of antibiotic resistance from a bacterial perspective.. Drug Resist Updat.

[63] Mariam DH, Mengistu Y, Hoffner SE, Andersson DI (2004). Effect of rpoB mutations conferring rifampin resistance on fitness of *Mycobacterium tuberculosis*.. Antimicrob Agents Chemother.

[64] Borman AM, Paulous S, Clavel F (1996). Resistance of human immunodeficiency virus type 1 to protease inhibitors: selection of resistance mutations in the presence and absence of the drug.. J Gen Virol.

[65] Leigh Brown AJ, Frost SD, Mathews WC, Dawson K, Hellmann NS (2003). Transmission fitness of drug-resistant human immunodeficiency virus and the prevalence of resistance in the antiretroviral-treated population.. J Infect Dis.

[66] Lu J, Sista P, Giguel F, Greenberg M, Kuritzkes DR (2004). Relative replicative fitness of human immunodeficiency virus type 1 mutants resistant to enfuvirtide (T-20).. J Virol.

[67] Fohl LM, Roos DS (2003). Fitness effects of DHFR-TS mutations associated with pyrimethamine resistance in apicomplexan parasites.. Mol Microbiol.

[68] Nagaev I, Bjorkman J, Andersson DI, Hughes D (2001). Biological cost and compensatory evolution in fusidic acid-resistant *Staphylococcus aureus*.. Mol Microbiol.

[69] Walliker D, Hunt P, Babiker H (2005). Fitness of drug-resistant malaria parasites.. Acta Trop.

[70] Hayward R, Saliba KJ, Kirk K (2005). pfmdr1 mutations associated with chloroquine resistance incur a fitness cost in *Plasmodium falciparum*.. Mol Microbiol.

[71] Kublin JG, Cortese JF, Njunju EM, Mukadam RA, Wirima JJ (2003). Reemergence of chloroquine-sensitive *Plasmodium falciparum* malaria after cessation of chloroquine use in Malawi.. J Infect Dis.

[72] Mita T, Kaneko A, Lum JK, Bwijo B, Takechi M (2003). Recovery of chloroquine sensitivity and low prevalence of the *Plasmodium falciparum* chloroquine resistance transporter gene mutation K76T following the discontinuance of chloroquine use in Malawi.. Am J Trop Med Hyg.

[73] Mita T, Kaneko A, Lum JK, Zungu IL, Tsukahara T (2004). Expansion of wild type allele rather than back mutation in pfcrt explains the recent recovery of chloroquine sensitivity of *Plasmodium falciparum* in Malawi.. Mol Biochem Parasitol.

[74] Wang X, Mu J, Li G, Chen P, Guo X (2005). Decreased prevalence of the *Plasmodium falciparum* chloroquine resistance transporter 76T marker associated with cessation of chloroquine use against *P. falciparum* malaria in Hainan, People's Republic of China.. Am J Trop Med Hyg.

[75] De-quan L, Rui-jun L, Dao-xing R, Da-qing G, Chun-yong Z (1995). Changes in the resistance of *Plasmodium falciparum* to chloroquine in Hainan, China.. Bulletin of the World Health Organization.

[76] Thaithong S, Suebsaeng L, Rooney W, Beale GH (1988). Evidence of increased chloroquine sensitivity in Thai isolates of *Plasmodium falciparum*.. Trans R Soc Trop Med Hyg.

[77] Abdel-Muhsin AM, Mackinnon MJ, Ali E, Nassir el KA, Suleiman S (2004). Evolution of drug-resistance genes in *Plasmodium falciparum* in an area of seasonal malaria transmission in Eastern Sudan.. J Infect Dis.

[78] Babiker HA, Satti G, Ferguson H, Bayoumi R, Walliker D (2005). Drug resistant *Plasmodium falciparum* in an area of seasonal transmission.. Acta Trop.

[79] Thanh NV, Cowman AF, Hipgrave D, Kim TB, Phuc BQ (2001). Assessment of susceptibility of *Plasmodium falciparum* to chloroquine, quinine, mefloquine, sulfadoxine-pyrimethamine and artemisinin in southern Viet Nam.. Trans R Soc Trop Med Hyg.

[80] Huong NM, Hewitt S, Davis TM, Dao LD, Toan TQ (2001). Resistance of *Plasmodium falciparum* to antimalarial drugs in a highly endemic area of southern Viet Nam: a study *in vivo* and *in vitro*.. Trans R Soc Trop Med Hyg.

[81] Nguyen MH, Davis TM, Cox-Singh J, Hewitt S, Tran QT (2003). Treatment of uncomplicated falciparum malaria in southern Vietnam: can chloroquine or sulfadoxine-pyrimethamine be reintroduced in combination with artesunate?. Clin Infect Dis.

[82] Rosario VE, Hall R, Walliker D, Beale GH (1978). Persistence of drug-resistant malaria parasites.. Lancet.

[83] Shinondo CJ, Lanners HN, Lowrie RC, Wiser MF (1994). Effect of pyrimethamine resistance on sporogony in a *Plasmodium berghei*/*Anopheles stephensi* model.. Exp Parasitol.

[84] Peters JM, Chen N, Gatton M, Korsinczky M, Fowler EV (2002). Mutations in cytochrome b resulting in atovaquone resistance are associated with loss of fitness in *Plasmodium falciparum*.. Antimicrob Agents Chemother.

[85] Wyllie AH (1980). Glucocorticoid-induced thymocyte apoptosis is associated with endogenous endonuclease activation.. Nature.

[86] Oberhammer F, Wilson JW, Dive C, Morris ID, Hickman JA (1993). Apoptotic death in epithelial cells: cleavage of DNA to 300 and/or 50 kb fragments prior to or in the absence of internucleosomal fragmentation.. Embo J.

[87] Muller A, Hacker J, Brand BC (1996). Evidence for apoptosis of human macrophage-like HL-60 cells by *Legionella pneumophila* infection.. Infect Immun.

[88] Garrity MM, Burgart LJ, Riehle DL, Hill EM, Sebo TJ (2003). Identifying and quantifying apoptosis: navigating technical pitfalls.. Mod Pathol.

[89] Abend M, Schmelz HU, Kraft K, Rhein AP, van Beuningen D (1998). Intercomparison of apoptosis morphology with active DNA cleavage on single cells *in vitro* and on testis tumours.. J Pathol.

[90] Peters GJ, van Triest B, Backus HH, Kuiper CM, van der Wilt CL (2000). Molecular downstream events and induction of thymidylate synthase in mutant and wild-type p53 colon cancer cell lines after treatment with 5-fluorouracil and the thymidylate synthase inhibitor raltitrexed.. Eur J Cancer.

[91] McGuire JJ (2003). Anticancer antifolates: current status and future directions.. Curr Pharm Des.

[92] Jackman AL, Calvert AH (1995). Folate-based thymidylate synthase inhibitors as anticancer drugs.. Ann Oncol.

[93] Curtin NJ, Harris AL, Aherne GW (1991). Mechanism of cell death following thymidylate synthase inhibition: 2′-deoxyuridine-5′-triphosphate accumulation, DNA damage, and growth inhibition following exposure to CB3717 and dipyridamole.. Cancer Res.

[94] Ayusawa D, Shimizu K, Koyama H, Takeishi K, Seno T (1983). Accumulation of DNA strand breaks during thymineless death in thymidylate synthase-negative mutants of mouse FM3A cells.. J Biol Chem.

[95] Yin MB, Guimaraes MA, Zhang ZG, Arredondo MA, Rustum YM (1992). Time dependence of DNA lesions and growth inhibition by ICI D1694, a new quinazoline antifolate thymidylate synthase inhibitor.. Cancer Res.

[96] Nippon Gene/Wako 2009–2010 Catalogue.. http://www.wako-chem.co.jp/siyaku/info/life/article/gene2007.htm.

[97] Jacobson MD, Weil M, Raff MC (1997). Programmed cell death in animal development.. Cell.

[98] Kerr JF, Winterford CM, Harmon BV (1994). Apoptosis. Its significance in cancer and cancer therapy.. Cancer.

[99] Bursch W, Kleine L, Tenniswood M (1990). The biochemistry of cell death by apoptosis.. Biochem Cell Biol.

[100] Arends MJ, McGregor AH, Wyllie AH (1994). Apoptosis is inversely related to necrosis and determines net growth in tumors bearing constitutively expressed myc, ras, and HPV oncogenes.. Am J Pathol.

[101] Howie SE, Sommerfield AJ, Gray E, Harrison DJ (1994). Peripheral T lymphocyte depletion by apoptosis after CD4 ligation *in vivo*: selective loss of CD44- and ‘activating’ memory T cells.. Clin Exp Immunol.

[102] Arnot DE, Gull K (1998). The *Plasmodium* cell-cycle: facts and questions.. Ann Trop Med Parasitol.

[103] Newbold CI, Boyle DB, Smith CC, Brown KN (1982). Stage specific protein and nucleic acid synthesis during the asexual cycle of the rodent malaria *Plasmodium chabaudi*.. Mol Biochem Parasitol.

[104] Leiden Malaria Research Group (LMRG). *P. berghei* - Model of malaria.. http://www.lumc.nl/con/1040/81028091348221/810281121192556/811070740182556/.

[105] Schetters TP, Hermsen CC, Van Zon AA, Eling WM (1988). Stage-specific proteins of *Plasmodium berghei*-infected red blood cells detected by antibodies of immune mouse serum.. Parasitol Res.

[106] Franke-Fayard B, Fonager J, Braks A, Khan SM, Janse CJ (2010). Sequestration and tissue accumulation of human malaria parasites: can we learn anything from rodent models of malaria?. PLoS Pathog.

[107] Kempe DS, Akel A, Lang PA, Hermle T, Biswas R (2007). Suicidal erythrocyte death in sepsis.. J Mol Med.

[108] Lang KS, Duranton C, Poehlmann H, Myssina S, Bauer C (2003). Cation channels trigger apoptotic death of erythrocytes.. Cell Death Differ.

[109] Lang KS, Lang PA, Bauer C, Duranton C, Wieder T (2005). Mechanisms of suicidal erythrocyte death.. Cell Physiol Biochem.

[110] Deponte M, Becker K (2004). *Plasmodium falciparum*–do killers commit suicide?. Trends Parasitol.

[111] Lines JD, Wilkes TJ, Lyimo EO (1991). Human malaria infectiousness measured by age-specific sporozoite rates in Anopheles gambiae in Tanzania.. Parasitology.

[112] Wu Y, Wang X, Liu X, Wang Y (2003). Data-mining approaches reveal hidden families of proteases in the genome of malaria parasite.. Genome Res.

[113] Kim R, Tanabe K, Uchida Y, Emi M, Inoue H (2002). Current status of the molecular mechanisms of anticancer drug-induced apoptosis. The contribution of molecular-level analysis to cancer chemotherapy.. Cancer Chemother Pharmacol.

[114] Bhowmick IP, Kumar N, Sharma S, Isabelle Coppens I, Jarori GK (2009). *Plasmodium falciparum* enolase: stage-specific expression and sub-cellular localization.. Malar J.

